# Cases from the Note Book of Service in the Medical Department of the U. S. Army

**Published:** 1871-05

**Authors:** Charles M. Clark

**Affiliations:** Late Surgeon 39th Ill. Vols.


ARTICLE IV.—*Cases from the Note Book of Service in the Medical Department of the U. S. Army.* By CHARLES M. CLARK, M.D., late Surgeon 39th Ill. Vols.

Two interesting cases of sudden death commencing at the heart.

First: Calcareous degeneration and deposit.

Second: Ossification and growth of bone.

CASE I.

*History of Case.* Solomon Blank, private 176th Penn. Vol. Infantry, aged 35 years, with large, muscular adipose development —formerly a brewer by occupation. Had been sick in regimental hospital at St. Helena Island, S. C., for some ten days, with an attack of acute rheumatism, as the assistant surgeon in charge informed us, and he had been treating the case with tr. aconiti radicis (fort.) in full doses, for several days, and in fact up to the time of death, which was very sudden and unexpected by him.

On the 20th day of February, 1862, Dr. Forbes, of the 67th Ohio Vols., and myself, were ordered by the Medical Director to make post-mortem examination of the body in order to ascertain the cause of death, for the reason that many of the officers and men of the regiment were disposed to think that the doctor had poisoned the deceased, and were threatening him with violence.

*Autopsy.* Six hours after death. The body was well nourished and presented fine physical conformation. The examination was chiefly directed to the organs of the thoracic region. The lungs were found adherent in many places to the costal pleura, the result of old inflammations, otherwise they were healthy.

The heart was found *in situ*, but one-third larger than normal. The pericardium contained an excess of fluid, and was adherent on all sides, and especially so, to the diaphragm. All the vessels leading from the right side of heart were undergoing the process of calcification, and the descending vena cava was especially remarkable for the extent of calcareous deposit, so arranged that it bore a striking resemblance to the trachea with its circular rings.

Within the endocardium of the right auricle was found a chalky mass the size of a large peanut, and resembling it in shape.

Other and smaller deposits were found in the substance of the right ventricle. The left side of heart was found hypertrophied and full of clots. The walls of the right ventricle were thin and completely studded with points of calcareous deposit.

This large deposit weighed seventy-three grains. The abdominal viscera was not examined, as it was thought sufficient cause for death had been found.

It is scarcely necessary to remark that the immediate cause of death was paralysis of the heart's function by the excessive use of tinct. aconite rad., given in full dose every third hour and continued for the space of four days. The remote cause of death was certainly plain enough, and gave us sufficient reason for exonerating the medical officer.

We afterwards learned that the deceased had been subject to acute attacks of rheumatism, more or less, for the past fifteen years. He was a German by birth, a brewer by occupation, and a great beer drinker. What connection there is between excessive use of malt liquor and chalky and ossific deposit within the heart, I will leave your readers to reflect upon after the narration of—

CASE II.

*History of Case.* Theodore Lott, private Co. F., 39th Ill. Vol. Infantry, age 44 years, German by birth, 5 feet, 8½ inches in height, light complexion, blue eyes, sandy hair, weight 196 pounds, and formerly a brewer by occupation.

June 4th, 1864, he was detailed for picket duty at the front, at a time when our advance was within six miles of Richmond, and remained on duty until the evening of June 5th, when the company was relieved from duty. While returning to their quarters, the enemy opened a brisk fire and they were double-quicked to camp, a distance of three-quarters of a mile.

He had complained during the day of not feeling very well, and on reaching the intrenchments, fell down and immediately expired. He was buried the same evening by order of the acting surgeon in charge of the regiment, who reported his death as one from "heat apoplexy."

On learning the history of the case, I had the body exhumed on the morning of the 6th, and conveyed to the field hospital where I made the

*Autopsy* at 3 P. M., June 6th, 1864. The body was still flexible, no evidences of "rigor mortis." There was extensive ecchymosis of head, face, neck and abdomen. After making the usual incisions for displaying the organs of the thorax and turning up the sternum, I was surprised at the amount and degree of the adipose material which covered everything like an apron, and was fully one-eighth inch in thickness over pleura and pericardium. The pericardium contained some fluid, but not sufficient to be considered abnormal. The lungs were perfectly healthy. The heart was found in position but greatly enlarged. Applied ligatures to all the vessels and removed the organ. In making incision through the walls of right side of heart, I found the right auricle very thin and paper-like, with fatty degeneration of the muscular fibre. The ventricle was in the same condition, and I could penetrate the wall with my thumb and finger by merely pinching it up. The semi-lunar valves were imperfect, as also the mitral. The left auricle was found thin and soft; the left ventricle hypertrophied.

On making incision through the aorta, my knife came in contact with a hard bony substance, and on examination, I found a well organized bone, shaped similarly to the "os coccyx," and having its attachment to one of the sinuses of the aorta. It measured one and one-half inches in length and three-fourths of an inch in circumference at the bone. It tapered gradually upwards, being nodulated at its centre and again at its terminus, and was so placed that its extremity rested within the axis of the aorta. The aortic valves were undergoing ossification.

The organs of the abdominal cavity were in healthy condition with exception of the kidneys. The mesentery was loaded with adipose.

The immediate cause of death in this case was apoplexy of the heart, caused by this impediment in the aorta, and the condition of the aortic valves.

The heart became distended with blood from the impetus given the circulation by reason of the fright and violent exercise, and it being unable to dispose of the unusual influx, there resulted paresis of the muscular fibre, and death.

